# Protocol: fine-tuning of a Chromatin Immunoprecipitation (ChIP) protocol in tomato

**DOI:** 10.1186/1746-4811-6-11

**Published:** 2010-04-09

**Authors:** Martiniano M Ricardi, Rodrigo M González, Norberto D Iusem

**Affiliations:** 1 Departamento de Fisiología, Biología Molecular y Celular. IFIByNE-CONICET. Facultad de Ciencias Exactas y Naturales, Universidad de Buenos Aires, Argentina

## Abstract

**Background:**

Searching thoroughly for plant *cis*-elements corresponding to transcription factors is worthwhile to reveal novel gene activation cascades. At the same time, a great deal of research is currently focused on epigenetic events in plants. A widely used method serving both purposes is chromatin immunoprecipitation, which was developed for Arabidopsis and other plants but is not yet operational for tomato (*Solanum lycopersicum*), a model plant species for a group of economically important crops.

**Results:**

We developed a chromatin immunoprecipitation protocol suitable for tomato by adjusting the parameters to optimise *in vivo *crosslinking, purification of nuclei, chromatin extraction, DNA shearing and precipitate analysis using real-time PCR. Results were obtained with two different antibodies, five control loci and two normalisation criteria.

**Conclusion:**

Here we provide a chromatin immunoprecipitation procedure for tomato leaves that could be combined with high-throughput sequencing to generate a detailed map of epigenetic modifications or genome-wide nucleosome positioning data.

## Introduction

Emerging high-throughput methods and bioinformatics technologies have great potential to accelerate the discovery of specific DNA regulatory elements that interact with transcription factors (TFs). However, the vast majority of plant *cis*-elements corresponding to the majority of TFs are unknown [1], in part due to a lack of optimised experimental methods to be carried out prior to genome-wide data analyses.

By contrast, a great deal of research is currently focused on epigenetic events in plants. This topic is particularly interesting because in plants, unlike animals, acquired epigenetic changes can be transmitted to progeny since germ cells differentiate from somatic tissues present in an adult individual. In addition, stable patterns of gene expression necessary for differentiation and long-term adaptation can be mitotically and meiotically inherited in the form of active or silent epigenetic gene variants via mechanisms associated with chromatin structure [2]. In this respect the role of histone modifications is becoming increasingly appreciated.

Chromatin immunoprecipitation (ChIP) is a widely used procedure both to identify novel TF-target genes and to examine histone modifications. It is currently used in Arabidopsis [3] but is not yet developed for use with tomato (*Solanum lycopersicum*), which is considered a model plant species for a group of economically important crops such as potato, pepper and eggplant.

Tomato has a reduced genomic size (950 Mb), a short generation time and routine transformation technologies. Moreover, it shares the same haploid chromosome number and a high level of conserved genomic organisation with other Solanaceous plants [4]. Despite Arabidopsis being a model plant suitable for many purposes, it has a smaller gene repertoire than tomato (25,000 vs. 35,000) [5] as they belong to different families (Brassicaceae and Solanaceae, respectively) that diverged early in flowering plant evolution, ~150 million years ago [6]. Consequently, there are gene families that appear smaller in the Arabidopsis genome compared to tomato such as the MADS-box genes involved in development and fruit ripening [7], as well as gene families that are even absent such as the ASR gene family [8], which is associated with water stress.

Since current ChIP protocols and commercial kits that have been designed for or tested with other plant species [9] do not work for tomato tissues, our goal was to develop a reliable ChIP procedure for tomato. Therefore, we adjusted critical parameters of ChIP in order to optimise each successive step, particularly crosslinking and chromatin extraction.

## Materials and methods

### Plant material

Commercial tomato plants were grown under controlled environmental conditions with a photoperiod of 16 light hours and 8 dark hours and a mean temperature of 24°C. Only healthy five-week plants were used in all experiments.

### Confocal microscopy

An Olympus instrument model FV-300 was used. The software was Fluoview 3.3. The objective lens was 60× NA 1.4.

### Micrococcal nuclease digestion

Nuclei were purified following the ChIP protocol from steps 6 to 14 and washed twice with nuclei resuspension buffer by 10 min of centrifugation at 12,000 × g. Micrococcal nuclease (Worthington Biochemical Corporation, Lakewood, NJ, USA) digestion was performed in 100 ul for 20 min at 37°C. The enzymatic reaction was stopped by resuming the ChIP protocol from step 35 (proteinase K). The resulting DNA fragments were then extracted and precipitated according to steps 36-39.

### DNA physical shearing

A Branson Sonic Dismembrator 102C instrument was used to achieve the necessary high-intensity ultrasound.

### Antibodies

The antibodies used were of ChIP-grade quality and purchased from Abcam, Cambridge, UK (anti-H3: catalogue code # 12079; anti-H3K9 me2: catalogue code # 1220).

### Buffers

#### Extraction buffer 1

0.44 M sucrose

10 mM Tris-HCl, pH 8.0

5 mM β-ME

#### Extraction buffer 2

0.25 M sucrose

10 mM Tris-HCl, pH 8.0

10 mM MgCl_2_

1% Triton X-100

5 mM β-ME

1× protease inhibitor cocktail

#### Percoll extraction buffer

95% V/V Percoll

0.25 M sucrose

10 mM Tris-HCl, pH 8.0

10 mM MgCl2

5 mM β-ME

1× protease inhibitor cocktail

#### Nuclei resuspension buffer

10% Glycerol

50 mM Tris-HCl, pH 8.0

5 mM MgCl_2_

10 mM β-ME

1× protease inhibitor cocktail

#### Nuclei lysis buffer

50 mM Tris-HCl, pH 8.0

10 mM EDTA

1% SDS

1× protease inhibitor cocktail

#### ChIP dilution buffer

1.1% Triton X-100

1.2 mM EDTA

167 mM NaCl

16.7 mM Tris-HCl, pH 8.0

1× protease inhibitor cocktail

#### Low salt wash

20 mM Tris-HCl, pH 8.0

150 mM NaCl

0.1% SDS

1% Triton X-100

2 mM EDTA

#### High salt wash

20 mM Tris-HCl, pH 8.0

500 mM NaCl

0.1% SDS

1% Triton X-100

2 mM EDTA

#### LiCl wash

0.25 M LiCl

1% NP-40

1% sodium deoxicholate

1 mM EDTA

10 mM Tris-HCl, pH 8.0

#### TE buffer

10 mM Tris-HCl, pH 8.0

1 mM EDTA

#### Elution buffer

1% SDS

0.1 M NaHCO_3_

#### Micrococcal nuclease buffer

50 mM Tris-HCl, pH 8.5

5 mM Mg acetate

25% glycerol

### Protocol

#### Crosslinking

1- Harvest 3-4 g of healthy young leaves and cut them into 5-10 mm pieces (we used 3-5 week- old plants).

2- Place no more than 1 g of cut leaves into a Falcon tube and rinse two times with 50 ml of milliQ water by gently shaking.

3- Remove all water and submerge the leaves in 37 ml of 1% formaldehyde in cold extraction buffer 1 and vacuum infiltrate for 10 min. The solution will boil, and the leaves should appear translucent.

4- Add 2.5 ml of 2 M glycine, mix well and vacuum-infiltrate for 5 additional min to stop crosslinking.

5- Remove buffer and rinse twice with cold milliQ water. Remove excess water as thoroughly as possible with a paper towel.

#### Chromatin isolation

6- Grind the tissue to a fine powder with a pre-cooled mortar and pestle and liquid nitrogen. At this step, the samples can be combined but we recommend to grind 1 g at a time in the same mortar.

7- Resuspend the powder in 30-40 ml of cold extraction buffer 1 (see below for composition details). Unless otherwise specified, all of the following steps should be done at 0-4°C.

8- Filter sequentially through 80 and 11 μm nylon mesh.

9- Spin the filtered solution for 20 min at 2,880 × g.

10- Remove the supernatant and resuspend the pellet in 10 ml of extraction buffer 2 (see below for composition details).

11- Incubate for 10 min on ice to lyse chloroplasts and spin for 20 min at 2,100 × g.

12- Remove the supernatant and resuspend the pellet in 4 ml of extraction buffer 2 without Triton X-100.

13- Spin for 20 min at 2,100 × g and resuspend the pellet in 4 ml of Percoll extraction buffer.

14- Spin for 10 min at 12,000 × g.

15- Carefully take the upper phase and dilute it at least 5 times into nuclei resuspension buffer (for composition, see below).

16- Spin for 10 min at 12,000 × g.

17- Discard the supernatant and resuspend the pellet in 4 ml of nuclei resuspension buffer. At this step, the samples can be stored at -20°C

#### Nuclei lysis and DNA shearing

18- Spin for 10 min at 12,000 × g.

19- Resuspend the pellet in 0.5 ml of nuclei lysis buffer.

20- Sonicate chromatin for 10 sec, 5 times at 15% power setting to shear DNA into 200- to 1000-bp fragments.

21- Centrifuge 5 min at 21,000 × g to pellet debris.

#### Chromatin immunoprecipitation

22- Transfer the supernatant into a new tube and quantify the DNA using the Quan-It dsDNA Broad-Range Assay kit (Invitrogen, San Diego, CA, USA). Alternatively, a small amount of the chromatin extract (10-20 μl) can be quantified by conventional GeneQuant analysis following de-crosslinking and phenol/chloroform purification. The minimal amount of DNA required to continue is about 18 μg, sufficient for processing the negative control (5 μg), the positive control (5 μg), the tube with the biologically relevant sample (5 μg) and the INPUT (2 μg).

23- Block 40 μl of protein A/G Plus agarose beads (Santa Cruz Biotechnology, Santa Cruz, CA, USA) with sheared salmon sperm DNA (at 0.2 mg/ml final concentration) and 0.5 mg/ml BSA.

24- Split the chromatin sample (approx. 450 μl) into three tubes of equal volume (150 μl) and dilute 1:10 (up to 1.5 ml) in ChIP dilution buffer (see below for composition details).

25- Wash the blocked beads three times with 1 ml ChIP dilution buffer. Pellet beads by centrifuging for 5 min at 1,000 × g. After each wash, carefully pipette off and discard exactly 1 ml of supernatant in order to maintain the original bead volume. It is critical to use more beads than needed in order to compensate for pipetting errors and to ensure that the same volume is added to all tubes.

26- Mix chromatin samples with 40 μl of beads for at least 1 h with gentle shaking.

27- Pellet and discard the beads (plus non-specifically bead-bound chromatin) and combine the three supernatants (the so-called pre-cleared chromatin) into a Falcon tube.

28- Split the sample into tubes containing 5-10 μg of DNA each. Save 2-4 μg as an INPUT control. Always use the same amount of chromatin in each independent experiment. For the INPUT, follow all the incubations without adding any reagent until step 31.

29- Incubate overnight with 2 μl of undiluted antibodies or non-immune serum and then with 40 μl of new beads for at least 1 h with gentle shaking.

30- Pellet the beads and wash for 10 min sequentially with 1 ml of:

- Low salt buffer

- High salt buffer

- LiCl wash buffer

- TE (two washes)

(see below for detailed buffer compositions)

After the final wash, remove TE thoroughly.

#### Elution

31- Add 250 μl of freshly prepared elution buffer to dissociate the bead-bound complexes. Add the elution buffer to the INPUT tube.

32- Vortex briefly and incubate for 15 min at 65°C with gentle shaking.

33- Pellet the beads, carefully transfer the supernatant to a fresh tube and repeat the elution of the beads. Combine the two eluates. At this step, the samples can be stored at -20°C

#### Crosslinking reversal

34- Add 20 μl of 5 M NaCl to the eluate and incubate for 6 hr at 65°C to reverse the crosslinking. To prevent evaporation, completely submerge the samples in a water bath or use mineral oil if a dry block is used.

35- Add 10 μl of 0.5 M EDTA, 20 μl of 1 M Tris-HCl pH 6.8 and 1.5 μl of 14 mg/ml proteinase K to the eluate and incubate for 1 h at 45°C.

#### DNA recovery

36- Extract DNA with equal volume of phenol/chloroform/isoamyl alcohol. Centrifuge 5 min at 5,000 g to separate the phases (commercial DNA clean-up columns may alternatively be used).

37- Add 0.1 volume of 3 M sodium acetate pH 5.3 to the aqueous phase and precipitate with 0.7 volumes of isopropanol in the presence of tRNA (1 μg/ml final concentration). Centrifuge 20 min at 18,000 g.

38- Wash pellet with 300 ul of 70% ethanol. Centrifuge 5 min at 12,000 g. Air dry. Resuspend the DNA pellet in 50 μl of Tris-HCl pH 8 or TE supplemented with 10 μg/ml RNase A.

39- DNA is now ready for analysis by PCR.

### Key steps

#### Crosslinking and its reversal

**Figure 1:**
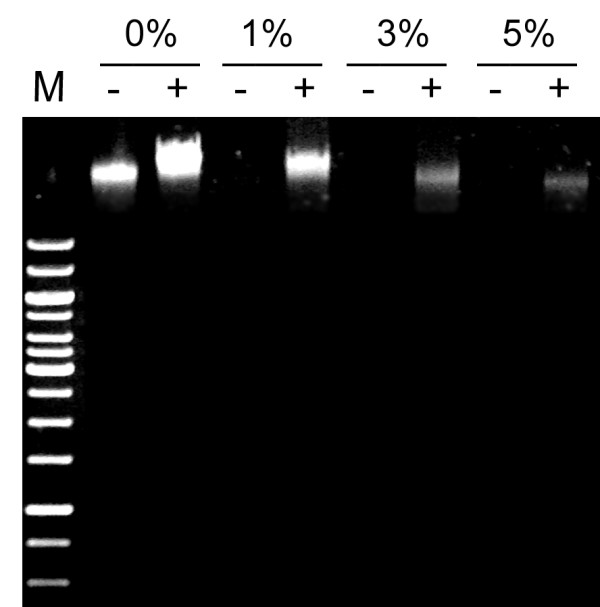
**Crosslinking efficiency**. Fresh leaves were vacuum infiltrated with buffer containing different formaldehyde concentrations. DNA was phenol/chloroform-extracted before (-) or after (+) crosslinking reversal with heat (65°C) and NaCl. M: lambda DNA cut by Hind III.

As Das et al. [10] highlighted, these steps are crucial. Tissue was vacuum infiltrated with different formaldehyde concentrations until it appeared translucent as air from mesophyll cells was replaced with the aqueous solution. The efficiency of crosslinking was evaluated by phenol extraction, taking advantage of the fact that only non-crosslinked DNA can be recovered in the aqueous phase. The optimal formaldehyde concentration to achieve efficient and reversible crosslinking turned out to be 1% (Figure 1). Too much formaldehyde was ineffective, probably because of poor crosslinking reversal and/or inhibition of chloroplast lysis as observed during the next step (see below), resulting in co-purification of nuclei along with chloroplasts and, thus, less pure chromatin (data not shown).

#### Chromatin extraction

**Figure 2:**
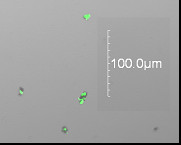
**Visualisation of nuclei**. Nuclei were stained with SYBR Green and observed using a confocal microscope (see Methods) at 60× through an oil immersion lens.

We combined the ChIP protocol for Arabidopsis [11] with previously described tomato nuclei isolation protocols [12,13]. The conditions involved filtration steps through nylon mesh, centrifugation in buffers of different density and incubation with the detergent Triton X-100 in order to lyse chloroplasts. Recovered intact nuclei were stained with SYBR Green and observed using a confocal microscope as 5-10 μm fluorescent particles (Figure 2).

**Figure 3:**
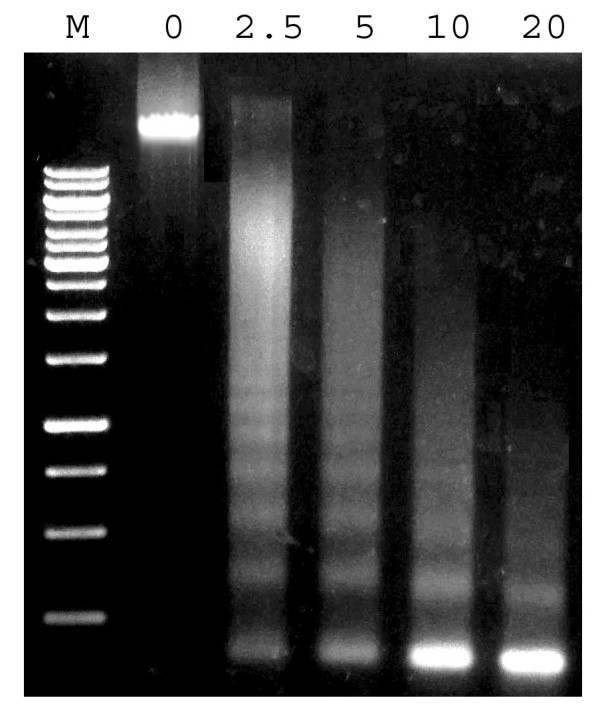
**Assessment of chromatin quality**. Non-crosslinked chromatin samples were digested for 20 min with 0, 2.5, 5, 10 and 20 units of micrococcal nuclease. M: 1-kb DNA ladder (MBI Fermentas, Inc.).

Once the integrity of the nuclei was confirmed, they were lysed in nuclei lysis buffer (see Methods for details) for subsequent chromatin isolation and shearing by sonication. Prior to the next step (shearing), chromatin quality was checked by micrococcal nuclease digestion. With this enzymatic treatment, non-crosslinked chromatin exhibited typical nucleosome ladders (Figure 3), demonstrating the quality of the samples. This offers the possibility of developing a protocol to perform native ChIP (NChIP). The pros and cons of NChIP vs. regular ChIP with crosslinking (XChIP) are discussed elsewhere [14].

#### DNA physical shearing

**Figure 4:**
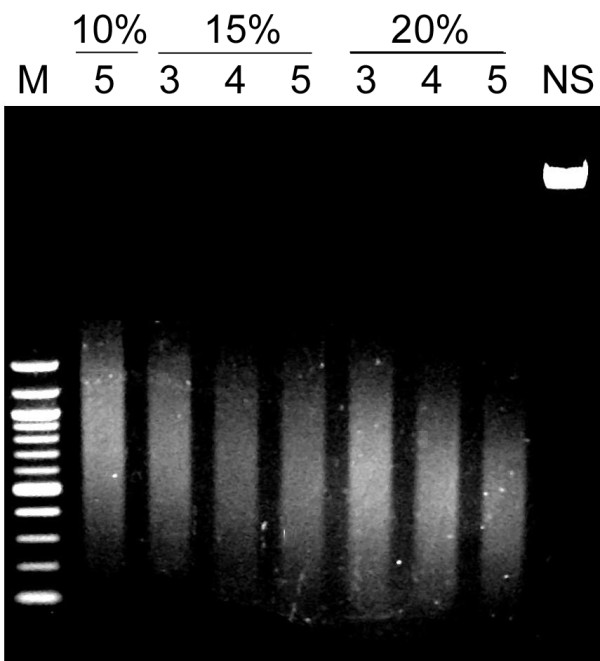
**DNA fragmentation**. Fresh chromatin samples were sonicated at 10, 15 or 20% power for 3, 4 or 5 pulses of 10 seconds each (waiting 5 seconds between pulses). NS: non-sonicated samples. M: "Quick load" 100-bp ladder (New England Biolabs, UK).

After testing various sonication conditions, we used 5 rounds of 10 seconds each at 15% amplitude, which was sufficient to obtain 200- to 1000-bp DNA fragments (Figure 4). Depending on the particular intended use of the ChIP technique, the average fragment size can be altered. For example, whereas larger fragments (1-2 kb) are recommended for cloning purposes, smaller fragments are suggested for high-resolution histone modification maps [15].

#### Immunoprecipitation

We used conditions essentially as previously reported [11]. We performed overnight incubation trials with ChIP-grade antibodies raised against Histone H3 (H3) or di-methyl-lysine 9 Histone H3 (H3K9 me2), an ubiquitous epigenetic mark in plants [16] and with non-immune serum as positive and negative controls, respectively.

#### Analysis of immunoprecipitated DNA

After crosslinking reversal and DNA extraction, we performed real-time (quantitative) PCR [17] to evaluate the amount of recovered DNA corresponding to the constitutively active genes ubiquitin (UBI) (GenBank: X58253) and Elongation Factor 1 (EF-1) (GenBank: X14449) [18] as well as the LTR-retrotransposons T135 (GenBank: AY746975) and ToRTL1 (GenBank: U68072), subregions To1 and To3 [19] (Table 1).

**Table 1:** DNA quantification by real-time PCR.

locus	forward primer	reverse primer	exponential slope
T135	CCAGCCATAACAACCAACTTC	GCAGACCACCAAATCCAACTC	1.93
To1	CCATCCTTTACTTCCATCATTG	ATCACATAGACCTCCTCGTTTC	1.99
To3	ATGAAGAGGAAGAAGAATACCG	TGGCAATGATGAGTGAAGAG	1.94
EF-1	GATTGGTGGTATTGGAACTGTC	AGCTTCGTGGTGCATCTC	1.93
UBI3	GCCGACTACAACATCCAGAAGG	TGCAACACAGCGAGCTTAACC	1.94

The locus-specific primers used and slope values resulting from standard curves using serial dilutions of INPUT samples [17] are indicated.

**Figure 5:**
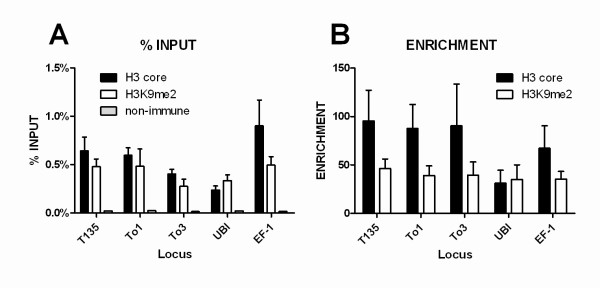
**Immunoprecipitated DNA analysis by real-time PCR**. Mean recovery values and standard errors of at least five replicates derived from three different chromatin isolation experiments. The indicated results were obtained from samples precipitated with anti-H3 (black closed bars), anti-H3K9 me2 (open bars) or non-immune serum (gray close bars, if noticeable). **A**: Results normalised relative to INPUT. **B**: Same results presented as enrichment relative to non-immune serum.

All precipitated specific DNA regions showed statistically higher recovery than the non-immune control (P = 0.0043 for total H3; P = 0.0025 for H3K9 me2), reaching values of up to 0.2-1% of the input sample (Figure 5A).

We also show the same results as the "relative enrichment", which is the enrichment relative to non-immune serum (Figure 5B). Although this data presentation format gives rise to high standard deviations, we were able to consistently observe more than 40-fold enrichment for the five loci precipitated with anti-total H3 and anti-H3K9 me2 antibodies. H3K9 me2/H3 coefficients did not differ significantly between the chosen loci (P < 0.05), strongly suggesting that the H3K9 methylation status of the LTR-retrotransposons, as well as UBI and EF-1, is similar.

As the finding of H3K9 Me2 in UBI and EF-1 housekeeping genes was unexpected (strictly based on Arabidopsis data [16]), further research on other genes will be needed to establish whether there are fundamental differences between Arabidopsis and tomato histone methylation patterns.

## Comments

Here we provide an optimised ChIP protocol for tomato samples to achieve unambiguous data interpretation. Results obtained with two different antibodies, five control loci and two normalisation criteria are shown. We believe that this procedure will allow both the identification of transcription factors targeting novel genes (given the availability of high-quality antibodies) and histone epigenetic analysis for genes of interest. Some modifications could clearly be introduced into this protocol in order to eventually carry out native ChIP in tomato or conventional ChiP in plants other than tomato. Since a reference tomato genome draft sequence is already available http://solgenomics.net/about/tomato_sequencing.pl, ChIP could be combined with high-throughput sequencing to generate a detailed map of epigenetic modifications or genome-wide nucleosome positioning data [20].

## Competing interests

The authors declare that they have no competing interests.

## Authors' contributions

MMR did the major experimental work. RMG contributed to the epigenetic context of the paper. NDI coordinated the project and drafted the manuscript. All authors read and approved the final manuscript.

## Authors' information

MMR and RMG hold doctorate fellowships from Consejo Nacional de Investigaciones Científicas y Técnicas (CONICET), Argentina. NDI is a member of the Carrera del Investigador Científico, CONICET, Argentina.
